# Molecular epidemiology and mechanisms of *Escherichia coli* resistance to ertapenem but not other carbapenems in China

**DOI:** 10.3389/fmicb.2026.1937582

**Published:** 2026-09-14

**Authors:** Dongliang Wang, Yuan Yuan, Wenjing Li, Junshuai Feng, Tianpeng He

**Affiliations:** 1 Department of Critical Care Medicine, Gansu Provincial Hospital, Lanzhou, Gansu Province, China; 2 Department of Critical Care Medicine, Longhua Hospital Affiliated to Shanghai University of Traditional Chinese Medicine, Shanghai, China; 3 Gansu University of Chinese Medicine, Lanzhou, Gansu, China

**Keywords:** ertapenem, *Escherichia coli*, OmpC, outer membrane protein, resistance

## Abstract

**Purpose:**

To investigate the molecular epidemiology and resistance mechanisms of carbapenem-resistant Enterobacteriaceae (CRE) with a specific phenotype resistant to ertapenem, but susceptible to meropenem and imipenem.

**Methods:**

*Escherichia coli* isolates (*n* = 424) were collected across the country; 54 isolates were CRE, of which 14 isolates were resistant to ertapenem but susceptible to meropenem and imipenem. Antimicrobial susceptibility testing were conducted using 17 drugs on the 14 isolates. Whole-genome sequencing (WGS) was used to analyze the isolates’ drug resistance genes carried, plasmid types, virulence genes, and drug resistance homology. Furthermore, efflux pumps and outer membrane proteins were analyzed.

**Results:**

Only 25.9% of the CRE isolates were resistant to ertapenem, and no carbapenemase genes were detected in any isolate. All 14 isolates carried the genes encoding extended-spectrum β-lactamase (ESBLs), and 8 *bla*_CTX-M_ subtypes were identified. Among these, *bla*_CTX-M-15_ (5 isolates) and *bla*_CTX-M-14_ (3 isolates) were the most prevalent. IncF plasmids (IncFIB, IncFIA, and IncFII) were the predominant plasmid types. Ten isolates simultaneously carried the highly virulent genes *iucC* and *iutA*. Efflux pump inhibition experiments with 14 isolates yielded negative results. Outer membrane protein analysis revealed that five of the isolates lacked OmpC. After genetic complementation with the outer membrane protein *ompC* gene, the minimum inhibitory concentration of ertapenem decreased 16–533 fold. Nine isolates with detectable OmpC expression also presented ertapenem monoresistance. Porin-coding gene disruption, *ramA/marA* gain-of-function mutations and efflux overexpression were excluded. Although these isolates harbored ESBL-encoding genes, ESBL alone could not confer ertapenem resistance. Potential mechanisms may include post-transcriptional porin repression, chromosomal *ompC* up-regulation or other permeability-altering chromosomal variants, and their precise mechanisms remain to be elucidated.

**Conclusion:**

These results suggest that the loss of outer membrane protein OmpC may be a key factor contributing to ertapenem resistance in carbapenemase-negative *E. coli* strains, and efflux pumps do not appear to mediate this resistance phenotype.

## 1 Introduction

*Escherichia coli* is the most common opportunistic pathogen causing both community and hospital-acquired infections. This bacterium can cause various diseases, including urinary tract, abdominal, and bloodstream infections. The long-term and large-scale clinical use of broad-spectrum antibacterial drugs has continuously exerted selective pressure on bacteria, leading to the common emergence and widespread dissemination of multidrug-resistant *E. coli* (Lasko and Nicolau, 2020). Carbapenems are considered last line of defense against severe infections caused by multidrug-resistant gram-negative bacteria (Hansen, 2021). Among these, ertapenem has a prolonged half-life, enabling once-daily administration; it is frequently selected for treating infections caused by ESBL-producing Enterobacterales. Given its narrow antimicrobial spectrum, ertapenem exerts lower selective pressure against *Pseudomonas aeruginosa* compared with antipseudomonal carbapenems (imipenem and meropenem) (Vasikasin et al., 2023); as such, it is the preferred drug for outpatient and emergency treatment of various infections (Wolie et al., 2025). However, in recent years, the rates of resistance of Enterobacteriaceae to ertapenem has significantly increased worldwide; these bacteria generally present with a specific resistance profile, with resistance to ertapenem but sensitivity to imipenem and meropenem (Wang et al., 2022). In China, the CHINET surveillance study conducted in the first half of 2025 revealed that *E. coli* had a resistance rate of 2.7% to ertapenem, which was higher than those to either meropenem (2.6%) and imipenem (2.4%).

The prevalence of carbapenem-resistant Enterobacteriaceae (CRE) in Saudi Arabia has shown imipenem, meropenem, and ertapenem resistance rates of 6.6, 9.1, and 18.6%, respectively (Alshehri and Irekeola, 2024).

The widespread prevalence of this drug-resistant phenotype has posed a significant challenge to clinical anti-infection diagnosis and treatment. The traditional screening and susceptibility testing systems for carbapenem resistance mostly use imipenem and meropenem resistance as the criteria for determining CRE, Notably, EUCAST recommends an ertapenem screening cut-off (MIC > 0.125 mg/L) for carbapenemase detection (Wang et al., 2024). This approach is prone to missing the detection of only ertapenem-resistant strains, thereby leading to the concealed spread of drug-resistant isolates. Meanwhile, these isolates do not exhibit typical carbapenem resistance characteristics and are often misdiagnosed as susceptible isolates in clinical, leading to treatment failure (Adelman et al., 2021). This special phenotype persists in clinical practice, causeing significant harm; however, the related drug resistance mechanisms have not been fully elucidated.

Mechanism of drug resistance in CRE primarily involves the production of various enzymes (extended-spectrum β-lactamase, AmpC enzyme, and carbapenemase), alterations in common binding sites for antibacterial drugs, enhanced expression of efflux pump components, and decreased membrane permeability caused by reduced expression of outer membrane proteins (OMPs) (Ma et al., 2023). Furthermore, the primary mechanisms underlying the resistance to carbapenems in CREs that do not produce carbapenemase are the production of ESBLs or AmpC β-lactamase binding (pAmpC) and overexpression of efflux pumps (Black et al., 2021). The outer membrane protein of *E. coli* is a key channel mediating the transmembrane uptake of carbapenem drugs (Zhou et al., 2023). However, the molecular mechanism underlying OmpC deficiency in the specific ertapenem resistance phenotype remains unclear. Moreover, studies on this type of strain remain limited; the existing literature mostly focuses on efflux pumps (Black et al., 2021). and relevant research on OMPs is lacking.

Therefore, this study collected data from 14 *E. coli* isolates classified as resistant to ertapenem, but susceptible to meropenem and imipenem, analyzing their molecular epidemiology and resistance mechanisms. We hypothesized that the absence of the outer membrane protein OmpC may be the core mechanism leading to the resistance of these strains to ertapenem. The results of this study will enhance our understanding of the mechanisms underlying carbapenem resistance and provide a theoretical basis for the rational use of antibacterial drugs in clinical settings.

## 2 Materials and methods

### 2.1 Clinical isolates

In total, 54 CRE isolates were identified from 424 *E. coli* strains isolated from 11 hospitals in China in 2023. *E. coli* identification was conducted using the VITEK®2 Compact System (bioMérieux, Lyon, France), and 14 isolates found to be resistant to ertapenem but susceptible to meropenem and imipenem were selected for further analysis. Detailed information about the strains and plasmids used in this study is presented in Table 1.

**Table 1:** Primers, strains and plasmids used in this study.

Strains and plasmids		
Strain or plasmid	Description	Source
ATCC25922	Quality control *Escherichia coli* strain used for minimum inhibitory concentration testing	Laboratory collection
DH5ɑ	*Escherichia coli*, competent cell strain used for transformation	TIANGEN Biotech
pHSG398	Plasmid, a gene replacement vector, Chloramphenicol	Laboratory collection
14 Isolates of *E. coli*	*E. coli* resistant to ertapenem but susceptible to meropenem and imipenem	Collected in clinic

Primers used in this study	
Primer name	Sequence 5′-3′
pHSG398-*ompC-SmaI*-F	aattcgagctcggtacccgggATGAAAGTTAAAGTACTGTCCCTCCTG
pHSG398-*ompC-XbaI*-R	tgcctgcaggtcgactctagaTTAGAACTGGTAAACCAGACCCAGA

### 2.2 Antimicrobial susceptibility testing

The minimum inhibitory concentrations (MICs) for a total of 17 antimicrobial agents, including ertapenem, imipenem, meropenem, tigecycline, polymyxin B, cefoxitin, cefepime, cefotaxime, amikacin, ceftazidime, piperacillin, ciprofloxacin, aztreonam, piperacillin-tazobactam, amoxicillin-clavulanic acid, Cefoperazone/sulbactam, and Trimethoprim/sulfamethoxazole were determined by broth microdilution. The breakpoints for all antibiotics were determined according to the CLSI M100-Ed36 (Clinical and Laboratory Standards Institute, 2026). The MIC for ertapenem was also determined in the presence of the efflux pump inhibitor carbonyl cyanide m-chlorophenylhydrazone (CCCP, 4 mg/L) to investigate the role of efflux pumps in carbapenem resistance, where a negative efflux-pump result was defined as a less than 4-fold reduction in ertapenem MIC for tested isolates following CCCP addition. (Ni et al., 2016). The carbapenemase phenotype was determined using the EDTA synergistic test, the enzyme inhibitor inhibition test and modified carbapenem inactivation method [mCIM]) yielded negative results. (Liao et al., 2021).

### 2.3 Whole-genome sequencing and analysis

Genomic DNA from overnight *E. coli* cultures was extracted with the TIANamp Bacteria DNA Kit (TIANGEN, Beijing, China) and qualified by agarose electrophoresis, Nanodrop (Thermo Fisher Scientific, Wilmington, DE, USA) and Qubit (Thermo Fisher Scientific, Wilmington, DE, USA). Hybrid ONT–Illumina NovaSeq whole-genome sequencing and genome assembly were performed by Shanghai Yuanxu Biotechnology Co., Ltd. Raw short reads were processed with FastQC v0.11.9 and Trimmomatic v0.39; genomes were assembled via Canu v1.8 and polished using Bowtie2 and Samtools, with genes predicted by Prodigal v2.6.2 and annotated against nr, CDD, KEGG, VFDB and CARD databases. Assembled contigs were analyzed on the Center for Genomic Epidemiology,^1^ accessed June 18, 2026, using ResFinder v4.1 and PlasmidFinder v2.1 to profile resistance genes, virulence factors, plasmids and MLST.

Meanwhile, this study extracted the full-length sequences of the outer membrane channel protein encoding genes *ompC*, *ompF* and their upstream promoters, as well as the outer transport regulation core genes *ramA* and *marA* from the assembled genomic sequences. These sequences were compared using the reference genome of *E. coli* K-12 MG1655 (accession: U00096.3) as a template.

### 2.4 Analysis of outer membrane proteins (OMPs)

OMPs were isolated and separated using sodium dodecyl sulfate-polyacrylamide gel electrophoresis (SDS-PAGE), as previously described (Carlone et al., 1986). In brief, the isolated proteins were loaded onto a 12% SDS-PAGE gel and electrophoresed for 25 min at 80 V and 50 min at 150 V (Bio-Rad, Hercules, CA, USA), followed by staining with 1% Coomassie brilliant blue (Beyotime, Beijing, China). Finally, the gels were exposed and photographed using a GelDoc XR + imaging system (Bio-Rad, USA) for protein band image acquisition.

### 2.5 Construction and complementation of plasmids carrying *ompC*

To investigate the effects of *ompC* deletion on ertapenem resistance, a plasmid encoding the functional *ompC* was constructed. First, the consensus *ompC* sequence was amplified using primers containing restriction endonuclease sites, and subsequently purified. Second, the amplified fragment and plasmid pHSG398, an arabinose-inducible chloramphenicol resistance vector, were double-enzyme digested to obtain the same cohesive termini; these were then ligated overnight with T4 ligase (Takara Bio Inc., Shiga, Japan) to generate the plasmid pHSG398-*ompC*. The recombinant plasmids were then used to transform competent cells *E. coli* DH5ɑ (Novagen, Darmstadt, Germany) via chemical conversion to obtain more successfully constructed plasmids to complement the wild strains. Successful transformants were identified by selection on Luria-Bertani agar containing 50 mg/L chloramphenicol and were confirmed by PCR and sequencing. The primers used to construct pHSG398-*ompC* are listed in Table 1. Finally, the extracted plasmid, pHSG398-*ompC* was electrotransformed (Dower et al., 1988) into an *ompC*-deficient clinical strain for subsequent analyses.

## 3 Results

### 3.1 MICs, antibiotic resistance profiles

A total of *E. coli* 424 isolates were obtained, of which 54 were classified as CRE, yielding an incidence rate of 12.7%. Among the 54 CRE isolates, 14, accounting for 25.9% of the total isolates, were only resistant to ertapenem, showing sensitivity to imipenem and meropenem. Antimicrobial susceptibility testing were conducted on these 14 isolates, showing that all were susceptible to tigecycline, polymyxin B, and piperacillin, but showed resistance to ciprofloxacin, cefoxitin, cefotaxime, and cefoperazone sulbactam. Other resistance rates were as follows: amikacin, 7.1% (1/14); cefepime and aztreonam, 92.8% (13/14); ceftazidime, 85.7% (12/14); trimethoprim-sulfamethoxazole, 78.6% (11/14). Finally, 10 isolates were susceptible to piperacillin-tazobactam, while 4 were intermediate; 7 isolates were resistant to amoxicillin-clavulanic acid, and 7 were intermediate (Figure 1).

**Figure 1:**
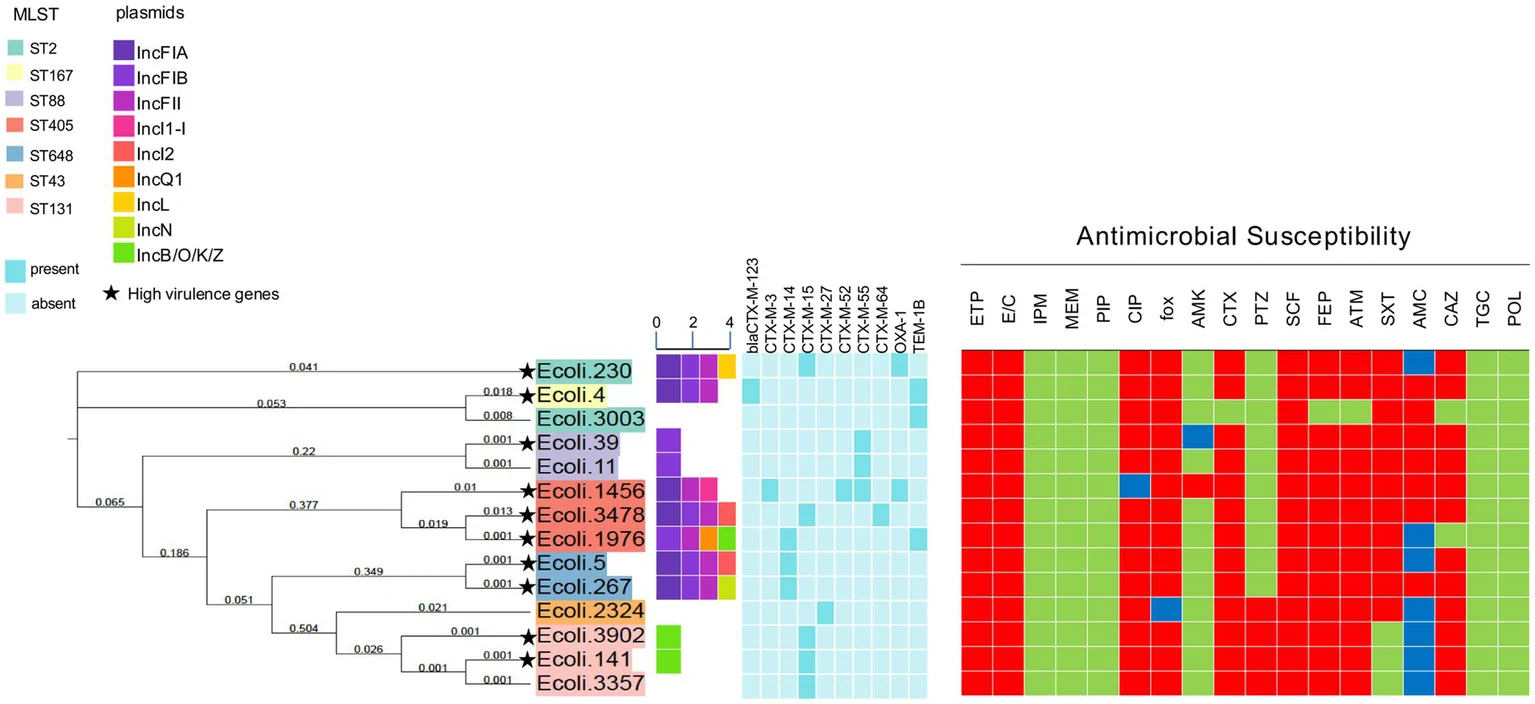
Tree diagram of 14 *E. coli* isolates that are only resistant to ertapenem based on phylogenetic trees, multilocus sequence typing, plasmids, high virulence, drug resistance gene profiles, and antimicrobial susceptibility testing profiles. MLST, Multilocus sequence typing; ETP, Ertapenem (S ≤ 0.5; I = 1; R ≥ 2); E/C, Ertapenem + carbonyl cyanide m-chlorophenylhydrazo; IPM, Imipenem (S ≤ 1; I = 2; R ≥ 4); MEM, Meropenem (S ≤ 1; I = 2; R ≥ 4); PIP, Piperacillin (S ≤ 16; I = 32–64; R ≥ 128); CIP, Ciprofloxacin (S ≤ 0.25; I = 0.5; R ≥ 1); Cefoxitin (S ≤ 8; I = 16; R ≥ 32); AMK, Amikacin (S ≤ 4; R ≥ 16); CTX, Cefotaxime (S ≤ 1; I = 2; R ≥ 4); PTZ, Piperacillin–tazobactam (S ≤ 8/4; R ≥ 32/4); SCF, Cefoperazone/Sulbactam (S ≤ 16; I = 32; R ≥ 64); FEP, Cefepime (S ≤ 2; R ≥ 16); ATM, Aztreonam (S ≤ 4; R ≥ 16); SXT, Trimethoprim–sulfamethoxazole (S ≤ 2/38; R ≥ 4/76); AMC, Amoxicillin Clavulanate (S ≤ 8/4; I = 16/8; R ≥ 32/16); CAZ, Ceftazidime (S ≤ 4; I = 8; R ≥ 16); TGC, Tigecycline (S ≤ 2; I = 4; R ≥ 8); POL, Polymyxin B (S ≤ 2; R ≥ 4). The results of the quality control strain ATCC 25922 in the antimicrobial susceptibility testing were all within the quality control range.

### 3.2 Analysis of drug resistance genes, plasmids, and virulence gene characteristics

No carbapenemase genes were detected in any of the 14 isolates. Consistently, all three phenotypic tests for carbapenemase production (EDTA synergistic test, enzyme inhibitor inhibition test, and modified carbapenem inactivation method [mCIM]) yielded negative results. Genes encoding β-lactamases were detected in 14 clinical isolates, among which eight CTX-M subtypes were detected (Figure 1). CTX-M-15 and CTX-M-14 were the most prevalent subtypes, detected in five and three isolates, respectively. In addition, TEM-1B (3 isolates) and OXA-1 (2 isolates) were detected. Five isolates carried multiple drug resistance genes simultaneously (Figure 1). Plasmid replicon typing was conducted on all isolates (Figure 1), revealing the following results: The IncF group (IncFIB, IncFIA, and IncFII) was the predominant plasmid type among the clinical *E. coli* isolates, with IncFIB, IncFIA, and IncFII detected in eight, six, and seven isolates, respectively. Several other replicon types, including IncI2, IncN, IncQ1, IncL, IncI1-I, and IncB/O/K/Z were also identified. Seven isolates harbored multiple plasmid replicons, while three isolates had no detectable plasmids. Ten isolates harbored both of the high-virulence genes iucC and iutA (Figure 1).

### 3.3 Multilocus sequence typing (MLST) and phylogenetic tree analysis

As presented in Figure 1, the 14 isolates were divided into seven types according to MLST results. ST131 and ST405 were the dominant STs, each comprising three isolates. ST2, ST648, and ST88 were represented by two isolates, while ST167 and ST43 were each represented by one isolate. The rooted phylogenetic tree constructed based on the core genome divided the 14 isolates of *E. coli* into five evolutionary branches, for which the branch lengths reflected the genetic differentiation level of the bacterial genomes of the strains. The ST2 type (*E. coli.*230) stands alone as a separate branch, showing the greatest genetic differentiation from the other isolates and the earliest evolutionary differentiation time. The ST167, ST88, and ST405 isolates all formed independent evolutionary subbranches. The fifth evolutionary branch included various isolates, including ST648, ST43, and ST131. Many isolates had extremely high homology and were classified in the same clonal group. The overall clustering results showed that the homologous MLST typed strains clustered into a single lineage branch, and the core genome evolution results were in agreement with MLST typing.

### 3.4 Role of OmpC in ETP resistance

To further clarify the potential resistance mechanisms of these isolates, we analyzed efflux pumps and outer membrane proteins in the 14 collected isolates. The efflux pump inhibition experiments on these 14 isolates showed negative outcomes (Figure 1). Further analysis of the outer membrane proteins of these 14 isolates (Figure 2A) revealed that five isolates lacked the outer membrane protein OmpC (Figure 2B). After supplementing the outer membrane protein pHSG398-*ompC* of these five isolates (Figure 2B), the MIC of ertapenem decreased 16 to 533 times (Table 2), indicating that among these five isolates, the absence of the outer membrane protein OmpC was the primary reason for the resistance of non-carbapenem-producing *E. coli* to ertapenem. Targeted WGS analysis of the 9 isolates with visible OmpC bands showed no protein-disrupting frameshift or nonsense mutations in *ompC* and *ompF* coding regions, supporting intact porin sequences. No gain-of-function mutations were detected in regulators *ramA* and *marA*.

**Figure 2:**
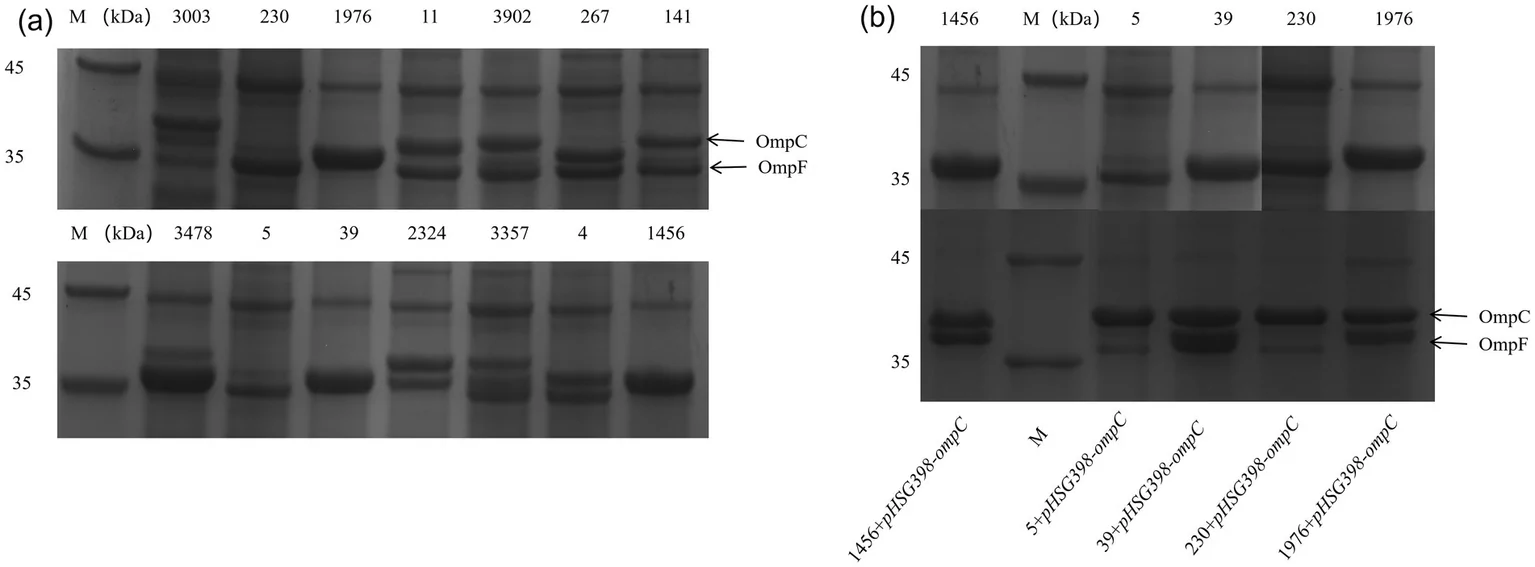
**(A)** The electrophoretic patterns of outer membrane proteins (OMP) of 14 *E. coli* isolates on a 12% sodium dodecyl sulfate-polyacrylamide gel. **(B)** The 5 *E. coli* isolates lacking OmpC and the corresponding strains with functional *pHSG398-ompC* supplementation were subjected to 12% SDS-PAGE analysis of their outer membrane protein.

**Table 2:** Drug sensitivity of wild isolates and clonal strains.

Isolates. No.	Clinical isolates		Functional replenishment *ompC* strains		MICs of ertapenem reductionmultiple
	Minimal inhibitory concentration (mg/L)				
	ETP	E/C	ETP	E/C	
*E. coli.*5	4	4	0.25	0.25	16
*E. coli.*39	>32	>32	0.06	0.06	533
*E. coli.*1976	8	8	0.5	0.5	16
*E. coli.*230	8	16	0.5	0.5	16
*E. coli.*1456	8	8	0.5	0.5	16

ETP, Ertapenem; E/C, Ertapenem + carbonyl cyanide m-chlorophenylhydrazo.

## 4 Discussion

The present study found that the resistance rate of *E. coli* to ertapenem was significantly higher than that to imipenem and meropenem. The number of ertapenem resistant strains is increasing every year; however, multicenter epidemiological and molecular mechanistic studies in China remain limited (Alshehri and Irekeola, 2024). This study identified 14 CRE isolates that were resistant only to ertapenem, with no detectable carbapenemase or AmpC-encoding enzymes. Current studies have shown that the production of ESBLs or AmpC enzymes, along with high expression of efflux pump systems, are the primary molecular mechanisms mediating ertapenem resistance in CRE isolates lacking the carbapenemase gene (Chetri et al., 2019). In this study, the efflux pump inhibition experiment was negative, indicating that efflux pumps were not associated with ertapenem resistance. All 14 isolates in this study carried ESBLs, of which 13 carried CTX-M-type ESBLs, with CTX-M-15 and CTX-M-14 being the predominant types. In addition, TEM-1B and OXA-1 were detected. However, the hydrolyzing ability of ESBLs alone is insufficient to increase the MIC of ertapenem to the resistance threshold, indicating the existence of a synergistic resistance mechanism (Tsai et al., 2013).

Outer membrane porins are crucial transmembrane channels that facilitate the penetration of β-lactam antibiotics into bacterial outer membranes. They are important structural components that affect intracellular drugs (Zhou et al., 2023). Our research group previously revealed that the absence of outer membrane proteins in *K. pneumoniae* leads to a high level of ertapenem resistance in strains that do not produce carbapenemases (Yuan et al., 2022). The SDS-PAGE results for the OMPs in this study showed that OmpC was absent in five isolates, while OmpC was normally expressed in the remaining isolates. After transferring the recombinant plasmid pHSG398-*ompC* into OmpC-deficient isolates and achieving functional restoration, the MIC of ertapenem decreased significantly by 16–533 times, confirming that the absence of OmpC only mediates resistance to ertapenem in carbapenemase-negative CRE isolates. The structural characteristics of carbapenem drugs explain this phenotype; ertapenem predominantly enters the outer membrane through the OmpC channel, whereas imipenem and meropenem can diffuse through other channels even when OmpC is dysfunctional (Tängdén et al., 2013). Previous studies have illustrated that mutations in the *ramA* can repress the expression of outer membrane porins and consequently induce ertapenem resistance. Nevertheless, no *ramA*-associated variants were detected in 9 isolates with intact OmpC expression. Furthermore, efflux pump inhibition assays returned negative results across all isolates, eliminating ertapenem resistance mediated by either *ramA*-driven porin down-regulation or efflux overexpression. Accordingly, the precise molecular mechanism responsible for ertapenem resistance in these nine isolates remains to be clarified in future investigations. Accordingly, the precise molecular mechanism responsible for ertapenem resistance in these nine isolates remains to be clarified in future investigations. Further quantitative transcriptional analysis of the ompC gene, especially in ertapenem-resistant isolates carrying intact functional ompC, is warranted to unravel the regulatory mechanisms contributing to this resistance phenotype in future investigations.

Overall, the present study described the epidemiological characteristics of ertapenem-resistant CRE in China. MLST data indicated that ST131 and ST405 were the dominant clones. Homologous transmission was observed among isolates. Eleven isolates harbored highly virulence genes, indicating that these isolates simultaneously face dual threats of multiple drug resistance and enhanced pathogenicity. Continuous targeted monitoring should be conducted to prevent outbreaks of high-risk clonal strains. Overall, the OmpC defect was the key permeability defect that led to the selective resistance of this isolate to ertapenem. Further studies are required to clarify the upstream signaling pathways regulating the expression of OmpC, as well as the mutation mechanisms that cause the loss of OmpC.

## Data Availability

The genome sequencing data have been deposited to BioProject accession numbers: PRJNA1511509, PRJNA1511512, PRJNA1511518, PRJNA1511528, PRJNA1511519, PRJNA1511537, PRJNA1511522, PRJNA1511531, PRJNA1511541, PRJNA1511544, PRJNA1511545, PRJNA1511520, PRJNA1511542, and PRJNA1511539 in the NCBI BioProject database.

## Footnotes

1
https://cge.cbs.dtu.dk/services/ResFinder/

## References

[ref1] Adelman M. W. Bower C. W. Grass J. E. Ansari U. A. Soda E. A. See I. . (2021). Distinctive features of ertapenem-mono-resistant carbapenem-resistant enterobacterales in the United States: a cohort study. Open Forum Infect. Dis. 9:ofab643. doi: 10.1093/ofid/ofab643 PMC8754373 35036469

[ref2] Alshehri A. A. Irekeola A. A. (2024). Prevalence of carbapenem-resistant enterobacterales (CRE) in Saudi Arabia: a systematic review and meta-analysis. Saudi Pharm. J. 32:102186. doi: 10.1016/j.jsps.2024.102186, 39498344 PMC11532984

[ref3] Black C. A. So W. Dallas S. S. Gawrys G. Benavides R. Aguilar S. . (2021). Predominance of non-carbapenemase producing carbapenem-resistant enterobacterales in South Texas. Front. Microbiol. 11:623574. doi: 10.3389/fmicb.2020.623574, 33643226 PMC7902696

[ref4] Carlone G. M. Thomas M. L. Rumschlag H. S. Sottnek F. O. (1986). Rapid microprocedure for isolating detergent-insoluble outer membrane proteins from haemophilus species. J. Clin. Microbiol. 24, 330–332. doi: 10.1128/jcm.24.3.330-332.1986, 3489731 PMC268907

[ref901] Chetri S. Bhowmik D. Paul D. Pandey P. Chanda D. D. Chakravarty A. . (2019). Acrab-tolc efflux pump system plays a role in carbapenem non-susceptibility in escherichia coli. Bmc Microbiol 19:210. doi: 10.1186/s12866-019-1589-1, 31488061 PMC6727511

[ref900] Clinical and Laboratory Standards Institute, (2026). Performance Standards for Antimicrobial Susceptibility Testing. 36th Edition, CLSI Supplement M100, Wayne, PA: Clinical and Laboratory Standards Institute.

[ref5] Dower W. J. Miller J. F. Ragsdale C. W. (1988). High efficiency transformation of e. coli by high voltage electroporation. Nucleic Acids Res. 16, 6127–6145. doi: 10.1093/nar/16.13.6127, 3041370 PMC336852

[ref6] Hansen G. T. (2021). Continuous evolution: perspective on the epidemiology of carbapenemase resistance among enterobacterales and other gram-negative bacteria. Infect. Dis. Ther. 10, 75–92. doi: 10.1007/s40121-020-00395-2, 33492641 PMC7954928

[ref7] Lasko M. J. Nicolau D. P. (2020). Carbapenem-resistant enterobacterales: considerations for treatment in the era of new antimicrobials and evolving enzymology. Curr. Infect. Dis. Rep. 22:6. doi: 10.1007/s11908-020-0716-3, 32034524 PMC7223591

[ref8] Liao Q. Yuan Y. Li Q. Wu S. Liu Y. Zhang W. . (2021). Comparing three different phenotypic methods for accurate detection of carbapenemase-producing enterobacterales. J. Infect Chemother. 27, 794–799. doi: 10.1016/j.jiac.2021.01.003, 33468425

[ref9] Ma J. Song X. Li M. Yu Z. Cheng W. Yu Z. . (2023). Global spread of carbapenem-resistant enterobacteriaceae: epidemiological features, resistance mechanisms, detection and therapy. Microbiol. Res. 266:127249. doi: 10.1016/j.micres.2022.127249, 36356348

[ref10] Ni W. Li Y. Guan J. Zhao J. Cui J. Wang R. . (2016). Effects of efflux pump inhibitors on colistin resistance in multidrug-resistant gram-negative bacteria. Antimicrob. Agents Chemother. 60, 3215–3218. doi: 10.1128/AAC.00248-16, 26953203 PMC4862505

[ref11] Tängdén T. Adler M. Cars O. Sandegren L. Löwdin E. (2013). Frequent emergence of porin-deficient subpopulations with reduced carbapenem susceptibility in esbl-producing *escherichia coli* during exposure to ertapenem in an in vitro pharmacokinetic model. J. Antimicrob. Chemother. 68, 1319–1326. doi: 10.1093/jac/dkt044, 23478794

[ref12] Tsai Y. Liou C. Fung C. Lin J. Siu L. K. (2013). Single or in combination antimicrobial resistance mechanisms of *Klebsiella pneumoniae* contribute to varied susceptibility to different carbapenems. PLoS One 8:e79640. doi: 10.1371/journal.pone.0079640, 24265784 PMC3827147

[ref13] Vasikasin V. Rawson T. M. Holmes A. H. Otter J. (2023). Can precision antibiotic prescribing help prevent the spread of carbapenem-resistant organisms in the hospital setting? JAC-Antimicrob. Resist. 5:dlad36. doi: 10.1093/jacamr/dlad036 PMC10050941 37008824

[ref14] Wang Y. Hu H. Shi Q. Zhang P. Zhao D. Jiang Y. . (2024). Prevalence and characteristics of ertapenem-mono-resistant isolates among carbapenem-resistant enterobacterales in China. Emerg. Microbes Infect. 13:2332658. doi: 10.1080/22221751.2024.2332658, 38517707 PMC10993752

[ref15] Wang D. Wang M. He T. Li D. Zhang L. Zhang D. . (2022). Molecular epidemiology and mechanism of *Klebsiella pneumoniae* resistance to ertapenem but not to other carbapenems in China. Front. Microbiol. 13:974990. doi: 10.3389/fmicb.2022.974990, 36425030 PMC9678918

[ref16] Wolie Z. T. Roberts J. A. López-Cortés L. E. Mirón-Rubio M. Pollard J. Marizan Nor A. N. . (2025). Current practices in outpatient parenteral antimicrobial therapy programmes: an international multi-Centre survey. Jac-Antimicrob. Resist. 7:dlaf75. doi: 10.1093/jacamr/dlaf075, 40433448 PMC12107060

[ref17] Yuan Y. Wang D. Cai H. Li D. Xu X. Guo Q. . (2022). High-level ertapenem resistance in *klebsiella pneumoniae* is due to ramA downregulation of *ompk35* through *micF*. Int. J. Antimicrob. Agents 60:106653. doi: 10.1016/j.ijantimicag.2022.106653, 35952849

[ref18] Zhou G. Wang Q. Wang Y. Wen X. Peng H. Peng R. . (2023). Outer membrane porins contribute to antimicrobial resistance in gram-negative bacteria. Microorganisms 11:1690. doi: 10.3390/microorganisms11071690, 37512863 PMC10385648

